# On the Efficacy of Animal Jelly in the Treatment of Intermittent Fevers; from the Account Given at the National Institute

**Published:** 1804-11-01

**Authors:** 


### On the Efficacy of Animal Jelly in the Treatment of Intermittent Fevers; *from the Account given at the National Institute, by Mr.* Halle.

Mr. Seguin having read before the National Institute, a memoir on the advantages of animal jelly considered as a febrifuge, it was determined that a committee should be appointed, in order to ascertain the fact, and to make some decisive experiments. Patients were accordingly admitted into a particular ward of the Hospital of Perfection (Hospice de Perfectionnement) of the School of Medicine. The mode of administering the remedy, and the details of the regimen, were solely regulated by Mr. Seguin himself. The intermittent fevers that were treated under the inspection of the committee, were twenty-two tertians, or tertianæ duplicatæ; fourteen quartans; ten quotidians; and twenty intermittents of an irregular type.

Amongst these patients, twenty were affected with prolonged longed autumnal fevers, eighteen with vernal fever, and six with new autumnal fevers; so that a great variety of circumstances took place in those experiments. The jelly had been prepared in the laboratory of the School of Medicine, and consisted of the best glue of Flanders, mixed with an equal quantity of sugar, dissolved in three or four times its weight of water; it was divided into square pieces, each of which was supposed to contain two drachms of pure jelly without the sugar or the water. The patients were ordered to take this jelly as well during the days of paroxysm as during those of intermission, in the morning, at noon, and in the evening. The dose amounted from three to six ounces of pure jelly; the diet consisted of roasted meat, half a litre (half a pint) of wine, and some prunes, and this meal was preceded by the use of a thick soup. Mr. Seguin, in general, ordered the patients to drink little, but allowed them a small quantity of brandy in the morning.

Of fifty-eight cases of intermittent fevers, on which the committee made their observations, fifty four terminated, after a greater or less number of fits, with an absolute or temporal cessation of the fever; this may either be ascribed to the action of the jelly, or be owing to the ordinary course of nature. Four fevers of the above kind, on the contrary, have resisted this treatment; two of the patients quitted the hospital without any relief; but the two others were cured, one by Peruvian bark, and the other by the use of ammonia, and of opium.

On the whole, the results which the committee have published are the following:

1. The diminution of the cold fit has been always so constant, and taken place so regularly in a number of patients, as to consider this phenomenon as affected by the jelly, which effect may likewise have advantageously influenced the termination of the fever itself.

2. The termination of the fever, though always preceded by the diminution of the cold fit, has, however, not been on an average proportionable to the first effect; as in general, it has not succeeded immediately, but proceeded in many patients so slowly, as to be evidently owing to the action of the jelly. This consideration, and the instances of the natural termination of fevers of this kind, render it uncertain which of the cases that fell under the committee's observations can be distinguished as being cured by the sole action of the jelly from those which may be owing to the power of nature only.

3. A

3. A certain number of cases have been observed, which were readily cured; others, in which the diminution of the symptoms has made constant progression till the absolute cessation of the fever; others again have come under the observation of the committee, in which an increase of the doses of the jelly has almost immediately produced a cessation of the fever, which therefore seems, in all probability, to be the result of the advantageous use of that remedy. These considerations, supported by about five and twenty detailed cases, lead us to suppose that each case affords an instance in which the phenomena of cure may be explained in favour of the great utility of animal jelly in these particular cases.

4. On comparing the effects produced by the jelly with the mode in which good Peruvian bark operates in removing fevers, when given in proper doses, and under convenient circumstances, it cannot be doubted that the efficacy of the jelly, such at least as has been observed by the committee, is by no means equal to that of good bark, as well with respect to the certainty, as to the perfection and continuance of effect with which the latter operates. One of the cases related by the committee proves, in a particularly evident manner, the difference in the action of the two remedies.

5. This observation likewise evinces, that in fevers which take or have the character of pernicious intermittents, and which are attended with imminent danger, the bark is a remedy for which the jelly can never serve as a substitute.

6. The action of the jelly is distinguished by some advantage, as being never followed by any inconvenient symptom, though it had been given in considerable doses, and sometimes continued longer than was necessary for determining its efficacy. It never produced any unfavourable symptom in fevers complicated with obstructions of the viscera, which makes us presume that it may be of use in such cases where bark is not given without inconvenience.

7. With respect to the cheapness of the jelly, compared to the price of the Peruvian bark, it may be stated, that the use of that substance is by no means so expensive, as it need not be continued for a long period; but when the fever prolongs itself, notwithstanding the administration of the jelly, as has been the case with a great number of patients, the expences attending the employment of this remedy are nearly as great, if not greater, than from Peruvian bark, particularly if the diet is put in calculation which is supposed to be necessary during the use of this mode of treatment.

8. The most obvious advantage which this remedy presents in the cases in which we may consider it as a febrifuge, consists in its being a remedy of easy preparation, which may always be at hand in places and under circumstances when it is impossible to obtain good Peruvian bark.

According to these observations, and the treatment executed under the eyes of the committee, the evident utility of the jelly may be stated as follows:

1. That is possesses the property of diminishing very sensibly the symptoms of the cold fit.

2. That its febrifuge action is not proved by evident facts, but rendered very probable by several instances.

Considering the importance of a discovery by which a common alimentary substance is proposed as an efficacious febrifuge, and observing that the experiments on which the conclusions of this account are founded, have not yet been made on a sufficient number of patients, nor continued during a time long enough for all circumstances of the different seasons, and all the varieties in which intermittent fevers appear, and for ascertaining the conditions under which this remedy may be serviceable, and the limits of its utility; the committee appointed for this purpose has proposed to the National Institute, that the continuation of those experiments might be ordered, and measures be taken for facilitating their execution, and for rendering them as complete and conclusive as can be desired.

